# Draft genome sequence data of *Nothopassalora personata,* peanut foliar pathogen from Argentina

**DOI:** 10.1016/j.dib.2024.110158

**Published:** 2024-02-06

**Authors:** Joaquin H. Monguillot, Renee S. Arias, Valerie A. Orner, Alicia N. Massa, Victor S. Sobolev, Nelson Bernardi Lima, Juan Paredes, Claudio Oddino, Marcelo Carmona, Cinthia Conforto

**Affiliations:** a Instituto de Patologia Vegetal, Centro de Investigaciones Agropecuarias, Instituto Nacional de Tecnología Agropecuaria, IPAVE-CIAP-INTA, Av. 11 de Septiembre, Córdoba 4755, Argentina; b Unidad de Fitopatologia y Modelizacion Agricola, Consejo Nacional de Investigaciones Científcas y Técnicas, UFyMA-CONICET, Av 11 de Septiembre, Córdoba 4755, Argentina; c United States Department of Agriculture (USDA), Agricultural Research Service (ARS), National Peanut Research Laboratory (NPRL), 1011 Forrester dr. S.E., Dawson, GA, USA; d Facultad de Ciencias Agrarias, UNCA, Av. Belgrano 300, Catamarca, Argentina; e Facultad de Agronomía y Veterinaria, IMICO, Ruta Nacional 36 km 601, Río Cuarto, Córdoba, Argentina; f Facultad de Agronomía, Universidad de Buenos Aires, Cátedra de Fitopatología, Av. S. Martín, Buenos Aires 4453, Argentina

**Keywords:** Late leaf spot, *Arachis hypogaea*, *Cercosporidium personatum*, MAT locus, Groundnut, Foliar disease

## Abstract

Late leaf spot (LLS) caused by the Ascomycete *Nothopassalora personata* (N.p.) (Syn. *Cercosporidium personatum*) is the main foliar disease of peanuts in Argentina and in peanut producing areas of the world, causing up to 70% yield losses. The extremely slow growth of this fungus in culture, that takes around one month to form a 1 cm colony (0.45 mm/day), and the lack of adequate young tissues from where to extract nucleic acids, have hindered genetic studies of this pathogen. Here, we report the first genome sequence of a *N. personata* isolate from South America, as well as genetic variants on its conserved genes, and the complete sequence of its mating-type locus MAT1-2 idiomorph. The *N. personata* isolate IPAVE 0302 was obtained from peanut leaves in Córdoba, Argentina. The whole genome sequencing of IPAVE 0302 was performed as paired end 150 bp NovaSeq 6000 and *de novo* assembled. Clean reads were mapped to the reference genome for this species NRRL 64463 and the genetic variants on highly conserved genes and throughout the genome were analyzed. Sequencing data were submitted to NCBI GenBank Bioproject PRJNA948451, accession number SRR23957761. Additional Fasta files are available from Harvard Dataverse (https://doi.org/10.7910/DVN/9AGPMG and https://doi.org/10.7910/DVN/YDO3V6). The data reported here will be the basis for the analysis of genetic diversity of the LLS pathogen of peanut in Argentina, information that is critical to make decisions on management strategies.

Specifications Table

**Table:** 

Subject	Genomics
Specific subject area	Fungal Plant Pathogen Genomics
Data format	Genome Sequencing, Raw data, De novo Assembly, Filtered Reads, Analyzed sequences
Type of data	Tables, Figures
Data collection	*Nothopassalora personata* was isolated from peanut (*Arachis hypogaea*) leaves of cv. Granoleico sampled in 2019, in Rio Cuarto, Córdoba, Argentina. Whole genome was sequenced on Illumina NovaSeq 6000 at the Genomics Center of the Univ. of California Davis, CA. Data were processed using CLC Genomics Workbench 23.0.4 (Qiagen, Aarhus, Denmark). Clean reads ≥ 140 nt were de novo assembled, and the assembly used to detect the MAT1-2 idiomorph locus of IPAVE 0302. Clean reads were also mapped to the genome reference NRRL 64463 and genome-wide distribution of Single Nucleotide Polymorphisms (SNP) quantified.
Data source location	*N. personata* isolate was collected from Cordoba, Argentina, 32° 24′ 30.5028″ S, 63° 42′ 18.9468″ W. Sequencing data were placed in public repositories: NCBI GenBank and Harvard Dataverse.
Data accessibility	Bioproject PRJNA948451, accession number SRR23957761. Repository name: National Center for Biotechnology Information (NCBI), and Harvard Dataverse Data identification number: (https://doi.org/10.7910/DVN/9AGPMG and of https://doi.org/10.7910/DVN/YDO3V6) Direct URL to data: https://www.ncbi.nlm.nih.gov/sra/SRR23957761

## 1 Value of the Data

• This is the first report of a whole genome sequence of *Nothopassalora personata* from South America.
• This genome shows many genetic variants when compared to the isolate NRRL 64463 from the United States. This information is critical to study the population genetics and evolution of the pathogen, and relevant to the development of disease management strategies.
• This is the first report of the complete MAT1-2 idiomorph sequence of *Nothopassalora personata*, and its putative recombination sites in this species, as it was detected on isolates IPAVE 0302 and NRRL 64463.
• The MAT locus affects sexual reproduction in fungi, and therefore their genetic diversity. The data reported here will be critical for genetic studies that focus on the biology of the pathogen.
• The slow growth of *N. personata* in laboratory has hindered the study of its biology and genetics.
• The data described here provide substantial information for comparative genomics and evolution of the species.

## 2 Background

Argentina is the first-largest peanut producing and exporting country in South America with 336,817 ha cultivated area concentrated in the central-south region of Cordoba province [1,2]. The crop is affected by two important aerial fungal diseases, early leaf spot (ELS) and late leaf spot (LLS) (*Passalora arachidicola* (Hori). LLS, etiological agent *Nothopassalora personata* Berk. &M.A. Curtis (Syn. *Cercosporidium personatum*, teleomorph *Mycosphaerella berkeleyi* W.A.Jenkins) is considered more aggressive, difficult to control and present world-wide. Frequent fungicide applications to control this pathogen can lead to the emergence of resistance, often associated with mutations in fungicide target genes. Having genome sequencing information is of vital importance as it allows the study of regions and mechanisms associated with fungicide resistance for subsequent more effective pathogen management.

Currently, there is no available information on the genome of *N. personata* for any peanut-producing country in the Southern Hemisphere. The purpose of this work was to obtain a draft genome of an isolate, IPAVE 0302, from the peanut producing area from Argentina to be used as a research model to understand the biology of this pathogen and its potential genetic diversity which could affect the strategies of disease control.

## 3 Data Description

The draft genome sequence of isolate IPAVE 0302 (accession number SRR23957761) is available at the National Center for Biotechnology Information (NCBI) under the BioProject number PRJNA948451. Statistics are summarized in Table 1. Variants were detected in conserved genes normally used in phylogenetics, including ribosomal RNA cistron, RNA polymerase II largest subunit (RPB1) and RNA polymerase II second largest subunit (RPB2), using the same parameters as for genome-wide variant detection. Three variants were found on the 18S rRNA, three on the 28S rRNA, and one variant each in RPB1 and RPB2, Table 2.

**Table 1:** Genomic features and de novo assembly statistics for *Nothopassalora personata* IPAVE 0302.

Attribute	Value
Genome size	34 306 332 bp
Number of contigs	1979
Largest contig (bp)	656 997
Average contig length (bp)	17 312
Coverage sequencing	1548 X
Number of scaffolds	1979
N25 (bp)	145 344
N50 (bp)	67 924
N75 (bp)	29 655
Overall GC content	51.91%

**Table 2:** Genetic variants observed on conserved genes of *Nothopassalora personata* IPAVE 0302 and the reference genome NRRL 64463 using a threshold of 35% of the reads. Nucleotides in parentheses “()” were only observed at 15% threshold.

	Ribosomal RNA cistron							RPB1	RPB2
Locus	18S	ITS1	5.8S	ITS2	28S	28S	28S		
Position (nt)	23	55-56	1852	2108	8098	8315	8502	4869	1377
NRRL 64463	C/G	- - -	- - -	(C/T)	- - -	- - -	- - -	- - -	- - -
IPAVE 0302	- - -	CG/GT	C/T	C/T	C/T	T/C	A/C	C/T	A/G

**Fig. 1:**
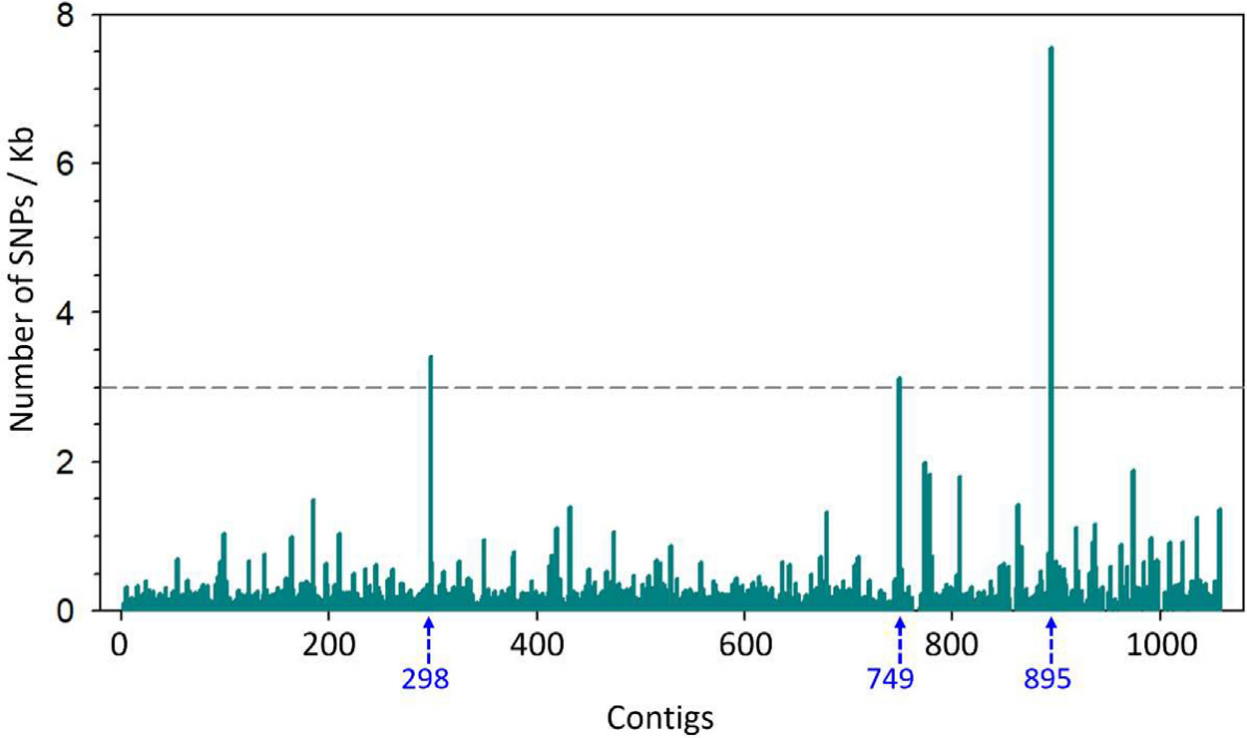
Genome-wide distribution of SNPs observed in IPAVE 0302 and not present in the reference genome, y-axis: SNPs per kilobase pair (kb), x-axis: contig numbers correspond to the location of SNPs using as reference the genome NRRL 64463.

**Fig. 2:**
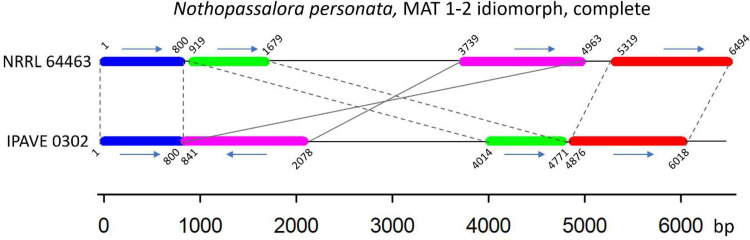
Schematic representation of the complete MAT1-2 idiomorph locus of *Nothopassalora personata* IPAVE 0302 compared to NRRL 64463, showing putative recombination.

Mapping of IPAVE 0302 genome sequencing reads to the 1061 contigs of the reference genome NRRL 64463, followed by calculation of number of single nucleotide polymorphisms (SNP) per kilobase were presented in Fig. 1. Examination of the MAT locus (Contig_298 in NRRL 64463), and further alignment to the reference genome showed potential recombination of this locus, Fig. 2.

## 4 Experimental Design, Materials and Methods

### 4.1 Fungal isolation

A monosporic culture of *Nothopassalora personata* was isolated from symptomatic peanut field of Rio Cuarto, Cordoba, Argentina, in 2019. The leaves were surface disinfected by submersion in a 1.5% NaOCl solution for 45 s and washed twice with sterile distilled water. The stromata, conidiophores and conidia were transferred to a Petri dish containing water agar (WA) and spread on the culture medium using a Drigalsky spatula. Single conidia germinated when observed with a stereomicroscope were transferred to plates with POA (peanut leaf extract + oatmeal agar) + D (dextrose) [3]. The plates were incubated for five-ten days at 24°C with a 12-h photoperiod. The isolate was preserved by Castellani's method [4] and deposited in the Culture Collection of the Instituto de Patología Vegetal – IPAVE (Córdoba, Argentina).

### 4.2 Genomic DNA preparation

Due to the slow growth of *N. personata*, genomic DNA was obtained from mycelium grown in liquid potato (200 g/L) + dextrose 10 g/L, (PD) medium + addition of 100 ml of the peanut leaf-extract (boiled 30 g peanut leaf cv. Granoleico/L) per 900 ml basal medium. After twelve days in a shaker incubator at 130 rpm, 24°C with a 12-h photoperiod [2], DNA was extracted using the Wizard® Genomic DNA Purification Kit (Promega Corp.). The concentration of DNA was determined using NanoDrop spectrophotometer (Thermo Scientific, USA).

### 4.3 Genome sequencing, assembly, and annotation

Two genomic DNA libraries were prepared from the isolate IPAVE 0302 using Illumina TruSeq Prep Kit V2 (Illumina, San Diego, CA), and sequenced as paired end 150 base pairs (bp) in Illumina NovaSeq 6000 System (Illumina, San Diego, CA) at the University of California Davis Genome Center (Davis, CA). Clean reads were generated by removing potential adapters, low quality reads, and those shorter than 140 bp using CLC Genomics Work bench 23.0.4 (Qiagen, Aarhus, Denmark). Clean reads were mapped to the 8511 ribosomal RNA operon of N.p., to the 5523 bp RNA polymerase II largest subunit gene (RPB1), and to the 4434 bp RNA polymerase II second largest subunit gene (RPB2) to corroborate taxonomic identification. A de novo whole-genome assembly was performed with clean reads ≥ 140 bp of both libraries, in addition, clean reads were mapped to the 1061 contigs of the reference genome *Nothopassalora personata* (N.p.) isolate NRRL 64463. Genetic variants, including insertions, deletions, single and multi-nucleotide polymorphisms, and potential amino acid (AA) changes were detected and compared to NRRL 64463 genome wide as well as on rRNA operon, RPB1, RPB2 [5]. All analyses were performed using CLC Genomics Workbench 23.0.4 (Qiagen, Aarhus, Denmark).

Mapping of IVAPE 0302 reads to the reference genome of N.p. NRRL 64463 showed the largest rates of genetic variants per kb in contig_298, contig_749 and contig_895, Fig. 1. Contigs 749 and 895 are shorter than 3 kb, however contig_298 is 59.5 kb long and contains a ∼6 kb region with homology to the mating-type locus MAT1-2 idiomorph of *Fulvia fulva* strain Race5_Kim (Syn. *Cladosporium fulvum*, Syn. *Passalora fulva*) (GenBank Accession: CP090175) published by [6]. Further analysis of the putative MAT1-2 locus showed structural differences when compared to NRRL 64463 genome reference, with an apparent rearrangement of genes and possible recombination that resulted in an inversion, Fig. 2. In other Ascomycetes, the MAT locus has been shown to play a role not only in sexual reproduction but also in pathogenesis [7]. In addition, sexual reproduction is a major source of genetic diversity of pathogens and can reduce the durability of host resistance [8]. Here, we report significant differences between the MAT1-2 locus of IPAVE 0302 isolated from Argentina and NRRL 64463 from U.S.A. The potential recombination in MAT1-2 locus will need to be considered when studying population genetics of *N. personata* in Argentina.

## Limitations

Not applicable.

## Ethics Statement

The current work does not involve human subjects, animal experiments, or any data collected from social media platforms.

## CRediT authorship contribution statement

**Joaquin H. Monguillot:** Methodology, Writing – original draft. **Renee S. Arias:** Conceptualization, Writing – original draft, Formal analysis, Data curation, Writing – review & editing. **Valerie A. Orner:** Formal analysis, Data curation. **Alicia N. Massa:** Formal analysis, Data curation, Writing – review & editing. **Victor S. Sobolev:** Writing – original draft. **Nelson Bernardi Lima:** Conceptualization, Methodology, Funding acquisition, Resources, Writing – review & editing. **Juan Paredes:** Methodology. **Claudio Oddino:** Methodology, Funding acquisition, Resources, Writing – review & editing. **Marcelo Carmona:** Funding acquisition, Resources, Writing – review & editing. **Cinthia Conforto:** Conceptualization, Methodology, Supervision, Writing – original draft, Funding acquisition, Resources, Project administration, Writing – review & editing.

## Data Availability

Replication Data for: Draft genome sequence data of Nothopassalora personata from Argentina (Original data) (Dataverse). Draft Genome of Cercosporidium personatum (syn. Nothopassalora personata) (Original data) (NCBI - GenBank). Replication Data for: Draft genome sequence data of Nothopassalora personata from Argentina (Original data) (Dataverse).
